# Pre-deployment assessment of an AI model to assist radiologists in chest X-ray detection and identification of lead-less implanted electronic devices for pre-MRI safety screening: realized implementation needs and proposed operational solutions

**DOI:** 10.1117/1.JMI.9.5.054504

**Published:** 2022-10-26

**Authors:** Richard D. White, Mutlu Demirer, Vikash Gupta, Ronnie A. Sebro, Frederick M. Kusumoto, Barbaros Selnur Erdal

**Affiliations:** a Mayo Clinic, Department of Radiology, Center for Augmented Intelligence in Imaging, Jacksonville, Florida, United States; b Mayo Clinic, Department of Cardiovascular Medicine, Jacksonville, Florida, United States

**Keywords:** artificial intelligence, MRI safety, pacemakers, loop recorders

## Abstract

**Purpose:**

Chest X-ray (CXR) use in pre-MRI safety screening, such as for lead-less implanted electronic device (LLIED) recognition, is common. To assist CXR interpretation, we “pre-deployed” an artificial intelligence (AI) model to assess (1) accuracies in LLIED-type (and consequently safety-level) identification, (2) safety implications of LLIED nondetections or misidentifications, (3) infrastructural or workflow requirements, and (4) demands related to model adaptation to real-world conditions.

**Approach:**

A two-tier cascading methodology for LLIED detection/localization and identification on a frontal CXR was applied to evaluate the performance of the original nine-class AI model. With the unexpected early appearance of LLIED types during simulated real-world trialing, retraining of a newer 12-class version preceded retrialing. A zero footprint (ZF) graphical user interface (GUI)/viewer with DICOM-based output was developed for inference-result display and adjudication, supporting end-user engagement and model continuous learning and/or modernization.

**Results:**

During model testing or trialing using both the nine-class and 12-class models, robust detection/localization was consistently 100%, with mAP 0.99 from fivefold cross-validation. Safety-level categorization was high during both testing (AUC≥0.98 and ≥0.99, respectively) and trialing (accuracy 98% and 97%, respectively). LLIED-type identifications by the two models during testing (1) were 98.9% and 99.5% overall correct and (2) consistently showed AUC≥0.92 (1.00 for 8/9 and 9/12 LLIED-types, respectively). Pre-deployment trialing of both models demonstrated overall type-identification accuracies of 94.5% and 95%, respectively. Of the small number of misidentifications, none involved MRI-stringently conditional or MRI-unsafe types of LLIEDs. Optimized ZF GUI/viewer operations led to greater user-friendliness for radiologist engagement.

**Conclusions:**

Our LLIED-related AI methodology supports (1) 100% detection sensitivity, (2) high identification (including MRI-safety) accuracy, and (3) future model deployment with facilitated inference-result display and adjudication for ongoing model adaptation to future real-world experiences.

## 1 Introduction

### 1.1 Lead-Less Implanted Electronic Devices: Categories/Types and Related MRI-Safety Issues

The significant incidence of lead-associated or generator pocket-related complications (e.g., infection) from the insertion of lead-dependent cardiac rhythm-management devices into the chest^1^ helped inspire the development of lead/generator-free versions, facilitated by progress with miniaturization, communications, and battery technologies.^2^ The outcome has been the creation of much smaller lead-less implanted electronic devices (LLIEDs) capable of (1) cardiac pacing or monitoring of (2) electrocardiographic activity; (3) cardiovascular physiology; (4) noncardiovascular chemistry (e.g., esophageal fluid pH^3^).^2^ Consequently, intrathoracic LLIED placement has become commonplace to meet various clinical needs.

As with any lead-dependent predecessor, the recognition of an LLIED’s presence, location, general category (e.g., pacing versus recording), and specific type (e.g., Micra™ versus Reveal LINQ™, respectively) is critical. The awareness of these factors can influence patient safety, LLIED function, clinical-support operations, and/or local environmental hazards. LLIED detection and identification are especially pertinent to the increasingly common electromagnetic and radiofrequency exposures during magnetic resonance imaging (MRI) examinations,^4^ including those being performed with systems functioning at increasing higher field strengths (e.g., 7 Tesla).^5^

Although most LLIEDs are considered “MRI conditional” (by posing no known hazards in a specified MRI environment with specified conditions of use),^6^ and despite some recent reports suggesting complete MRI safety at conventional field strengths (e.g., 1.5 and 3 Tesla),^7^ it remains imperative to acknowledge key facts. These include the following realities: (1) MRI conditional does not mean MRI compatible or safe, especially considering ever-changing MRI technology (e.g., increasing prevalence of 7-Tesla systems);^5^^,^^8^ (2) Not all MRI-conditional LLIEDs carry equivalent potential risks, partly due to the coexistence of other implants;^9^ (3) Even when considered MRI conditional, MRI exposure may result in recordable patient-related effects from an inserted LLIED or detectable alterations in LLIED function;^7^^,^^10^ (4) Some MRI-conditional LLIEDs are considered more stringently conditional than others;^11^ (5) Different MRI-conditional LLIEDs justify specific patient and/or LLIED assessment or preparation before and/or after, as well as monitoring during, the MRI examination;^6^^,^^12^^,^^13^ (6) Some LLIEDs are considered “MRI unsafe” (by posing a significant risk in all MRI environments).^3^^,^^11^^,^^12^^,^^14^

### 1.2 LLIEDs: Pre-MRI Screening

#### 1.2.1 General pre-MRI screening procedures

Accordingly, at an initial patient visit for an MRI examination, knowledge of an LLIED previously inserted at another institution is typically gained through direct interaction between the scanning supervisors (i.e., physician or technologist) and the patient (hopefully, possessing specific LLIED details). This is followed by manual entry of attained screening information into the patient’s electronic medical record (EMR).^6^^,^^12^^,^^13^ On the other hand, if the LLIED was intramurally placed, this information is likely gleaned by EMR review. Regardless, both forms of data extraction and documentation have known deficiencies for safety screening,^15^ thereby enabling an LLIED to remain inadequately recognized up to the time of (and possibly during) MRI scanning, especially in stressful emergency or trauma situations.^16^^,^^17^ Compounding LLIED-specific potential risks from MRI exposures are unpredictable causative factors related to patient or scanning differences.^4^^,^^6^^,^^9^^,^^12^ Other scenarios where LLIED recognition is also important include (1) External cardioversion (potential device malfunction and/or damage); (2) Radiation therapy (potential device malfunction and/or damage); (3) Cremation (potential device battery explosion).^18^^,^^19^

As mentioned, prerequisite patient and/or LLIED assessment or preparation (before, during, or after an MRI examination) may differ even when LLIEDs are considered MRI conditional. For example, when pertaining to an MRI-conditional lead-less pacemaker (LLP), the expectations typically include cardiologist-dependent (1) Pre-MRI evaluation of the patient and/or LLIED (likely necessitating LLP-setting adjustment); (2) Direct patient monitoring during MRI scanning; (3) Post-MRI evaluation of the patient and/or LLIED (with LLP resetting to original state).^6^^,^^12^^,^^13^ These demands exceed those when an MRI-conditional lead-less recorder (LLR) is involved, and precautions taken alone by the MRI technologist are deemed adequate.^6^^,^^12^^,^^13^ Therefore, the failure to differentiate between these two common MRI-conditional LLIED categories (i.e., “assessment-requiring” and “simple,” respectively) well before initiating MRI scanning could either put a patient at undue risk or disrupt operations (e.g., incorrect pre-examination readiness of supporting services, such as cardiology).

#### 1.2.2 Use of a chest X-ray in pre-MRI screening

A chest X-ray (CXR) is a standard component of pre-MRI safety screening (for LLIEDs or other man-made objects in the chest).^20^^–^^27^ Such CXR-based screening assumes even greater importance when there is inadequate EMR documentation from lack of a prior visit and/or internal misrecording.^26^^–^^28^ Unfortunately, any LLIED could be overlooked on a CXR due to their mutually small sizes (subject to projection-related distortions), especially when accompanied by (1) Suboptimal radiographic technique (e.g., under-penetration); (2) Patient-related factors (e.g., motion-related blurring); (3) Obscuration by adjacent-internal or superimposed-external radio-opaque or electronic materials. In addition, LLIED categories and/or types might be confused with each other by the interpreting radiologist because of (1) LLIEDs having remarkably similar appearances and positions on a frontal CXR (typically the only view acquired in emergency/trauma department or intensive care unit settings, without a lateral view, revealing LLIED intrathoracic location deep within the right ventricle for an LLP versus subcutaneous within the anterior chest wall for an LLR); (2) General lack of familiarity by a radiologist with LLIED-specific characteristics (especially retained legacy systems or recently introduced devices).^23^^,^^28^ These fundamental issues are especially germane to the less familiar, infrequently used, much smaller, and more “stringently” MRI-conditional LLIEDs [e.g., pulmonary artery pressure monitor (PAPM) for heart failure^20^^,^^24^^,^^29^] and MRI-“unsafe” LLIEDs [e.g., esophageal reflux capsule (ERC) for pH-monitoring^3^^,^^11^^,^^12^^,^^14^], which can easily go unnoticed.

### 1.3 Implanted Electronic Device Recognition on CXR: Potential Role for Artificial Intelligence

#### 1.3.1 Artificial intelligence: lead-dependent electronic device recognition on CXR

Other investigators have realized the potential value of CXR-reliant recognition of standard cardiac rhythm-management devices (including lead-dependent pacemakers and cardioverter-defibrillators), for which a comprehensive and detailed manual stepwise visual flowchart CARDIA-X system was initially proposed.^30^ More recently, an Artificial Intelligence (AI)-based system for CXR identification of lead-dependent devices (which routinely display radiographic text-based identifiers^31^) recognized the device manufacturer and type with 99.6% and 96.4% accuracy, respectively.^32^ However, the same AI model demonstrated a lower manufacturer-identification accuracy of 71% compared to another AI model running on either a mobile phone application or web platform^33^ (accuracy 89% and 73%, respectively), thereby approximating the nonAI-based CARDIA-X performance (i.e., accuracy 85%).^34^ None of the aforementioned studies or a very recently reported study of only lead-dependent pacemaker detection,^35^ focused on the recognition of the continuously evolving array of much-smaller modern LLIEDs (which do not display radiographic text-based identifiers).

#### 1.3.2 AI: opportunity for assisting radiologists in CXR-based LLIED recognition

Thus, AI-based assistance to radiologists in the prompt and confident frontal-CXR detection and localization of any general category of LLIED, and then the identification of its specific type, prior to a scheduled or urgent MRI could have significant safety and operational benefits. In response, our group previously developed a potentially high-performing cascading AI model, described technically elsewhere.^36^

Unlike the previous basic-technology phase of our research,^36^ this work focused on the pre-deployment assessment of our combined LLIED-detection and identification AI model for its current readiness, as well as the operational prerequisites to potentially assisting radiologists (reliably, effectively, and efficiently) once truly deployed in real-world clinical practice.^37^^,^^38^ The evaluations included: (1) Accuracies in the identification of each specific LLIED-type, and consequently the related MRI-safety level, based on experiences during both model development and simulated trialing;^39^^,^^40^ (2) Clinical MRI-safety implications of observed LLIED nondetections or misidentifications;^39^^,^^40^ (3) Anticipated (or unanticipated) infrastructure-architectural and/or workflow requirements for productive real-world clinical deployment;^41^^–^^43^ (4) Expectations and challenges related to ongoing model adaptation to changing real-world conditions.^44^^–^^46^

## 2 Methods

### 2.1 Original AI Model for LLIED Recognition

#### 2.1.1 Ground-truth LLIED-type labeling of CXR images

As previously detailed,^36^ Institutional Review Board-approved retrospective data-mining (spanning: March 1993 to February 2021) allowed the organization-wide extraction of digital CXR examinations (i.e., “AI model development population”) representing a wide range of LLIEDs supporting the development of an AI methodology for device detection followed by identification.^36^ The specific identities of the LLIED types represented, and their associated clinical implications, were not profiled in the previous nonclinical technical note.^36^

**Fig. 1:**
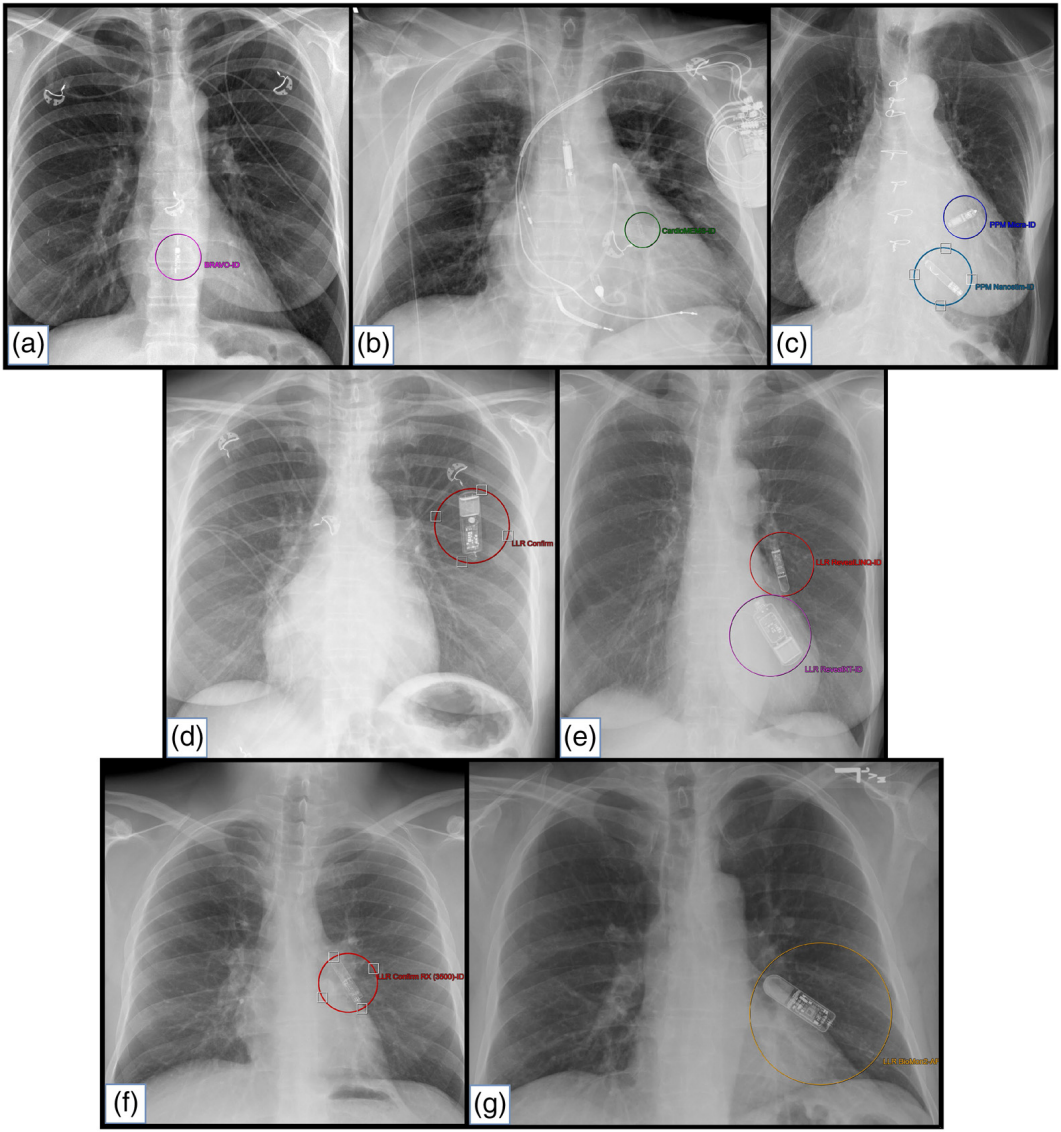
LLIED-type labeling. Using ROI-annotation capabilities of the GUI, examples of the nine LLIED types represented during the development of the original model are shown on frontal views. The types are arranged in order of decreasing levels of MRI-related risk and/or lessening requirements for patient or LLIED assessment (e.g., cardiology pre-, intra-, and post- MRI evaluation) as follows: (a) Unsafe ERC (Bravo™ Reflux Capsule); (b) Stringently Conditional PAPM (CardioMEMS™ HF); (c) Assessment-requiring conditional LLPs (Nanostim™* and Micra™); (d) Simple conditional LLR (CONFIRM™*); (e) Simple conditional LLRs (Reveal™ XT* and Reveal LINQ™); (f) Simple conditional LLR (CONFIRM Rx™); (g) Simple conditional LLR (BioMonitor2-AF). (* = Legacy LLIED no longer being implanted but possibly retained).

Serving as project “ground-truth” expert, a fellowship-trained cardiothoracic radiologist with 37 years of experience used a local graphical user interface (GUI)^36^^,^^47^ to manually delineate the specific LLIED type(s) demonstrated on a CXR image from the AI model development population.^36^ The frontal view (i.e., Postero–Anterior aka P–A, or Antero–Posterior aka A–P) from each CXR examination was correspondingly labeled using the interactive region-of-interest (ROI) capabilities of the GUI,^36^ with circular markers applied to derive square ROIs for input into model development (Fig. 1).^36^^,^^47^

**Fig. 2:**
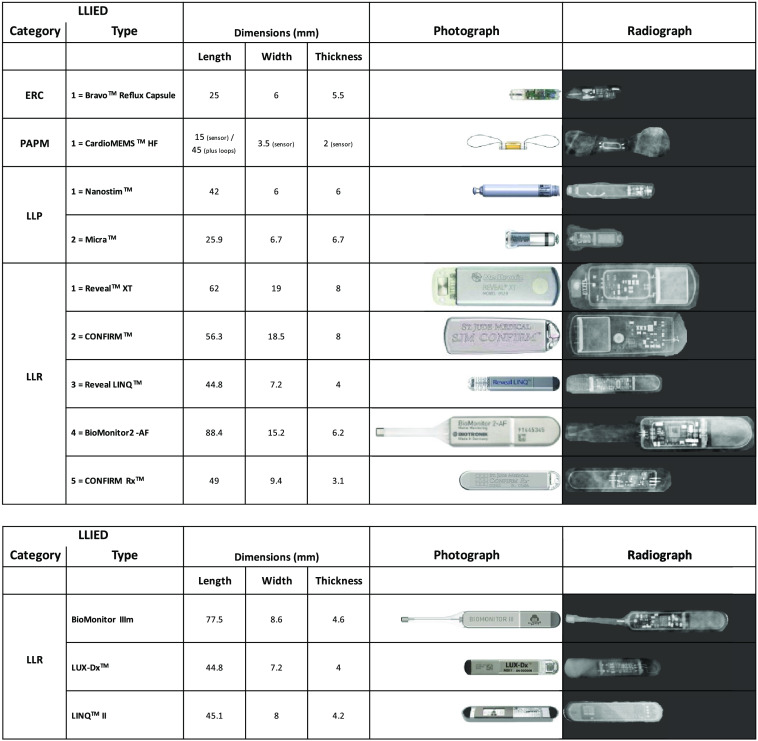
LLIED categories/types represented. LLIED-types in AI model development population (Above) and new types appearing in methodology trial population.

The LLIED categories, including ERC (one type), PAPM (one type), LLP (two types), and LLR (five types), denoted decreasing levels of MRI-related risk and/or lessening requirements for patient or LLIED evaluation (i.e., unsafe, stringently conditional, assessment-requiring conditional, or simple conditional, respectively) (Table 1 and Fig. 2).

**Table 1:** LLIED categories/types and MRI-safety levels represented.

LLIED		EMA/FDA	MRI safety^48^^–^^52^	
Category	Type	Approval	1.5 Tesla	3.0 Tesla
**Found in AI model development population or methodology trial population**				
**ERC**	1 = Bravo™ Reflux Capsule^a^	December 2010	Unsafe	Unsafe
**PAPM**	1 = CardioMEMS™ HF^b^	October 2006	Conditional**	Conditional**
**LLP**	1 = Nanostim™^b^^,^^c^	October 2013	Conditional*	Conditional*
	2 = Micra™ (M# MC1 VR01 or AVR1)^a^	April 2016	Conditional*	Conditional*
**LLR**	1 = Reveal™ XT (M# 9529)^a^^,^^c^	November 2007	Conditional	Conditional
	2 = CONFIRM™ (DM2102)^b^^,^^c^	August 2008	Conditional	*INA*
	3 = Reveal LINQ™ (M# LNQ11)^a^	February 2014	Conditional	Conditional
	4 = BioMonitor2-AF^d^	April 2016	Conditional	Conditional
	5 = CONFIRM Rx™ (DM3500)^b^	September 2017	Conditional	Conditional
**First presentation in methodology trial population**				
**LLR**	BioMonitor IIIm^d^	April 2020	Conditional	Conditional
	LUX-Dx™ (M# M301)^e^	June 2020	Conditional	Conditional
	LINQ™ II^a^	July 2020	Conditional	Conditional

*EMA* = European Medicines Agency.

ERC = Esophageal Reflux Capsule.

FDA = United States Food and Drug Agency.

LLIED = Lead-less implanted electronic device.

LLP = Lead-less pacemaker.

LLR = Lead-less recorder.

PAPM = Pulmonary artery pressure monitor.

Conditional = Simple conditional (safe if following specific recommendations or guidelines per manufacturer).

Conditional* = Assessment-requiring conditional (like simple but prerequisite patient and/or device assessment or preparation).

Conditional** = Stringently conditional (safe only if imaged under strict and highly specific technical restrictions).

Unsafe = Unsafe in any MRI environment.

*INA* = Information not available.

a Medtronic (Minneapolis, Minnesota).

b Abbott/St. Jude Medical (Little Canada, Minnesota).

c Legacy LLIED no longer being implanted but potentially retained.

d Biotronik SE & Co. (Berlin, Germany).

e Boston Scientific Corporation (Marlborough, Massachusetts).

During ROI labeling of instances of the original nine LLIED types on CXR images, a basic quality grade reflecting general conspicuity and detail clarity was applied as follows: (1) Unequivocally diagnostic supporting IDentification (“ID” in 76%); (2) Potentially nonrecognizable (“NR” in 12%) for detection or ID; (3) ID with superimposed or abutting materials, or incomplete inclusion within view margins, causing over-lapping (“OL” in 10%); (4) Combined (“NR and OL” in 2%).^36^

#### 2.1.2 LLIED-type recognition by original LLIED Model

To optimize data use from the AI model development population during training, validation, and testing of the nine-class “original LLIED model,” conventional approaches to data distribution, expansion, and augmentation (including LLIED-specific inclusion of labeled diagnostic lateral views: Table 2) were employed.^36^

**Table 2:** Criteria for LLIED lateral view exclusion from use in model development.

LLIED		Exclusion criteria
Type	Entity	
**ERC**	**1**	All included
**PAPM**	**1**	All included
**LLP**	**1**	Excessive foreshortening preventing:
		• Simultaneous visualization of fixation helix and distal battery chevron^53^^,^^54^ (and)
		• Appearance of body length > three times diameter
	**2**	Excessive foreshortening preventing:
		• Simultaneous visualization of cathode/tine complex and electronics-battery transition zone (∼0.5 body length)^55^ (and)
		• Appearance of body length > two times diameter
**LLR**	**1**	Excessive foreshortening preventing:
		• Simultaneous visualization of the battery-electronics transition zone (∼0.4 body length) and electronics-antenna transition in rectangle-shaped body^56^^–^^58^ (and)
		Lack of *en-face* presentation facilitating:
		• Visualization of rectangular distal electrode^56^^–^^58^
	**2**	Excessive foreshortening preventing:
		• Simultaneous visualization of the battery-electronics transition zone (∼0.4 body length) and electronics-antenna transition in slightly teardrop-shaped body^56^^,^^57^ (and)
		Lack of *en-face* presentation facilitating:
		• Visualization of triangular distal electrode^56^^,^^57^
	**3**	Excessive foreshortening preventing:
		• Simultaneous visualization of the battery-electronics transition zone (∼0.3 distance) and electronics-antenna transition in rectangle-shaped body^58^^,^^59^ (and)
		Lack of *en-face* presentation facilitating either:
		• Visualization of three-dot pattern aligned along electronics board and antenna base^58^^,^^59^ (or) Visualization of corrugated-appearing medradio antennae supporting cellular communication^58^^,^^59^
	**4**	Excessive foreshortening preventing:
		• Simultaneous visualization of the battery-electronic transition zone (∼0.4 body length) and faintly radio-opaque elongated antenna with distal electrode cap^60^^,^^61^ (and)
		Lack of *en-face* presentation facilitating:
		• Visualization of two small projections from body at base of antenna^60^^,^^61^
	**5**	Excessive foreshortening preventing:
		• Simultaneous visualization of battery-electronics transition zone (∼0.5 body length) and electronics-antenna transition in rectangle-shaped body^60^^,^^62^ (and)
		Lack of *en-face* presentation facilitating either:
		• Visualization of two projections to triangular antenna supporting Bluetooth communication (or) visualization of plaid-like pattern in battery^60^^,^^62^

**Fig. 3:**
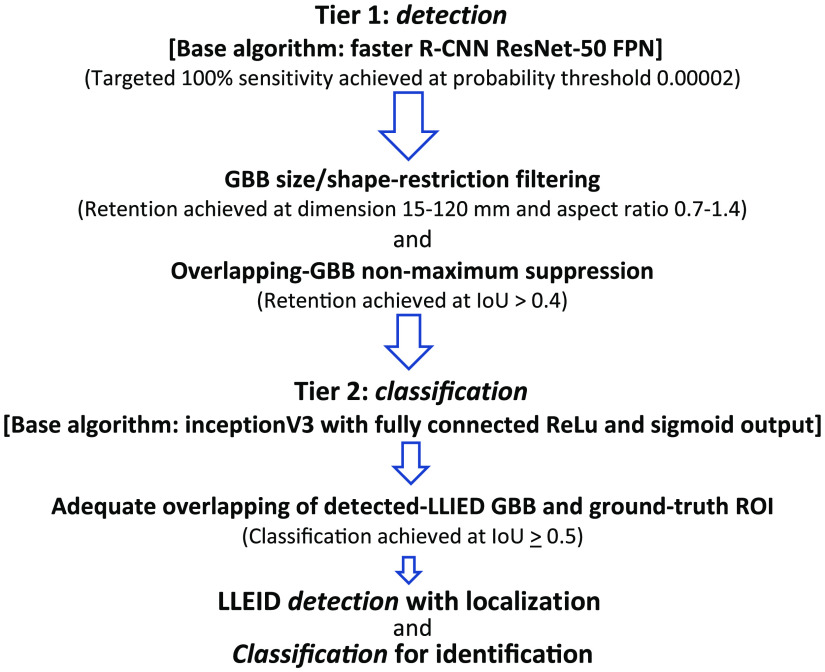
Two-tier cascading methodology and data flow for generic LLIED detection and classification^36^ (GBB, generated bounding box; IoU, intersection-over-union).

As previously detailed,^36^ a two-tier system underlying the original LLIED model for LLIED recognition was used: (1) First, to emphasize the detection of device presence and location; (2) Second, to support device-type identification, if detected and then classifiable.^36^ Ultimately, this prompted the creation of a cascading neural network methodology as follows (Fig. 3).

##### Tier 1: LLIED detection

For the detection with localization of any LLIED-related ROIs in the AI model development population, a faster region-based convolutional neural network (R-CNN) was used.^36^ Inherent to this method was the output of the inference results as generated bounding boxes (GBBs).^36^

Promoting a prerequisite to detect all LLIEDs and miss none, a probability-threshold reduction (i.e., to 0.00002) was needed to overcome suboptimal image quality and achieve 100% detection sensitivity in the validation dataset.^36^ The targeted detection sensitivity of 100% (i.e., recall value = 1.00) was maintained in testing, during which: (1) a true positive (TP) inference result was recorded when a GBB and a ground-truth LLIED-related ROI overlapped with intersection-over-union (IoU)≥0.5; (2) a false positive (FP) resulted from a GBB failing to overlap at IoU≥0.5; (3) a false negative (FN) resulted from a failure to create any GBB.^36^

##### Tier 2: LLIED identification—specific-type and related MRI-safety level

After theoretically achieving 100% device detection sensitivity in tier 1, a supposedly high generic device identification accuracy in tier 2 was previously described.^36^ With the combined goals of (1) Reducing FP results from tier 1; (2) Supporting maximal identification of specific LLIED types, all postfiltered (size/shape-based) detection-related GBBs (i.e., those overlapping with ground-truth ROIs at IoU≥0.5 in tier 1) were classified using a multiclass CNN.^36^ The network was then refined further using ground-truth ROIs initially for the nine-class classifier (per specific LLIED type); for the determination of correct LLIED-type identification, correspondence was confirmed by the GBB label resulting in the greatest IoU with a ground-truth LLIED-related ROI.^36^ Unlike the prior technical note,^36^ the LLIED identities and CXR appearances represented by the original nine classes, as well as newer classes, are presented in this report.

However, due to analytical restrictions from inadequate numbers of unique-patient instances for some LLIED types (typically legacy or newer types),^63^ a fundamental assessment of tier-2 accuracy in identifying MRI-safety level per LLIED category (i.e., unsafe, stringently conditional, assessment-requiring conditional, or simple conditional) was also performed.

### 2.2 Evaluation and Support of the Evolving LLIED Model for Device Recognition

Initially, during our “pre-deployment” work, three anticipated evaluations of the performance of the original LLIED model in LLIED recognition were completed within two populations (Table 3). However, three essential technical developments designed to overcome fundamental deployment workflow challenges facilitated both unanticipated early model evolution and a “limited-deployment” opportunity for repeat performance evaluation of an updated model in two other populations.

**Table 3:** LLIED model performance evaluations and essential technical developments.

**Original LLIED model: pre-deployment LLIED-recognition performance evaluations**
• Cross-validation assessment^a^
• Safety-level and specific-type identification accuracies during model testing^a^
• Basic pre-deployment trialing^b^
**Essential technical developments supporting real-world model deployment and adaptation**
• Selection/development of viewer for AI model inference-result display and adjudication
• Applying data standards, supporting interoperability, and enhancing user experience
• Preparation for ongoing adaptation of LLIED-detection and identification AI model
**Updated LLIED model: limited-deployment LLIED-recognition performance evaluations**
• Cross-validation assessment^c^
• Safety-level and specific-type identification accuracies during updated model testing^c^
• Limited-deployment simulated real-world trialing^d^

Patient population used:

a AI model development population.

b Methodology trial population.

c AI model update population.

d Updated methodology trial population.

**Fig. 4:**
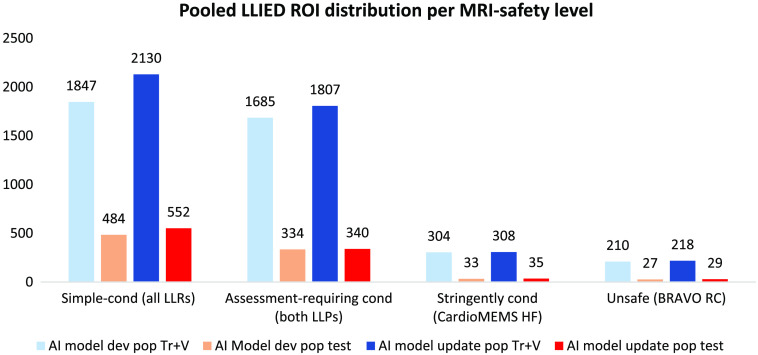
Pooling of testing ROI data of LLIED sets with matching MRI safety supported a fundamental four-category assessment of tier-2 safety-level identification accuracy. Pooling ultimately had no effect on either the stringently conditional or unsafe categories, because each was represented by a single specific LLIED type (Cond, conditional; LLP, lead-less pacemaker; LLR, lead-less recorder; Pop, population; Tr+V, training and validation; Test = testing).

The aforementioned fundamental assessment of tier-2 accuracy in identifying MRI-safety level was facilitated by pooling testing ROI data between LLIED sets with matching safety level (Fig. 4); the resulting four combined categories (per safety level) underwent accuracy assessment.

#### 2.2.1 Performance evaluations of original LLIED model for LLIED recognition

##### Cross-validation assessment

To further assess the pre-deployment durability of the original LLIED model,^36^ a fivefold cross-validation^64^ was executed on tier 1 for LLIED detection in the AI model development population (Table 3). However, corresponding cross-validation was not completed on tier 2 for either safety-level or specific-type identification, due to the recognized limitation of the approach when there are inadequate numbers of unique-patient instances (Fig. 4),^63^ as pertained to some types (especially legacy) in the AI model development population and expected with the initial appearances of new types in the future. For example, if there are very few (<5) patients representing an LLIED type, it is not feasible to perform cross-validation and report statistically significant and valuable results.

##### Safety-level and specific-type identification accuracies during model testing

Unlike the previous basic-technology report,^36^ the specific identities, photographic appearances, CXR delineations, and MRI-safety levels of the original nine classes of LLIEDs were tabulated and described in this work for future clinical application of the original LLIED model or newer versions (Table 1 and Fig. 2). The original LLIED model^36^ was initially assessed for its accuracy in identifying both MRI-safety levels (i.e., unsafe, stringently conditional, assessment-requiring conditional, or simple conditional) and specific type during model testing within AI model development population (Table 3). In addition, the clinical MRI-safety implications of any model-related nondetections or misidentifications of LLIEDs were reviewed.

##### Basic pre-deployment trialing

To help imitate a basic real-world trialing of the original LLIED model,^36^ a “methodology trial population” of 150 new randomly selected patients (not previously represented in the AI model development population) was compiled after additional data-mining (spanning: March 2021 to June 2021) (Table 3). From the methodology trial population: (1) The most recent frontal CXR image demonstrating any LLIED was collected from 100 LLIED patients; (2) One frontal CXR image was collected from 50 nonLLIED patients. The resulting 150 unannotated images (i.e., without prior ROI delineation by the ground-truth expert) underwent AI processing by our two-tier cascading original LLIED model for both LLIED detection and then LLIED-type identification via the automatic GBB-based display of AI inference results (returned in <1  s) using the aforementioned GUI.

After the AI-model processing, as previously described, the 100 unannotated LLIED-demonstrating frontal CXR images were manually labeled by the ground-truth expert using the GUI annotation capabilities^36^^,^^47^ while blinded to the previous model-generated inference results. Accordingly, 101 ROI labels (one LLIED case with two devices) were applied to indicate: LLIED presence/location, specific LLIED type, and ROI-quality grade (ID in 71 or 70%; NR in 15 or 15%; OL in 13 or 13%; NR and OL in 2 or 2%). Inference-result corroboration (again based on IoU≥0.5 and matching of LLIED labels between the model-derived GBB and the applied ROI)^36^ was then assessed.

#### 2.2.2 Essential technical developments supporting real-world model deployment and adaptation

A component-based simulation of deployment of our methodology for AI-based LLIED detection and identification on CXR was considered consistent with several recent FDA-endorsed actions.^44^ Hence, we pursued the following opportunities to facilitate the utilization of verified AI model output by the CXR-interpreting radiologist (Table 3; Appendix A).^40^^–^^44^^,^^65^^,^^66^

##### Selection/development of viewer for AI model inference-result display and adjudication

**Fig. 5:**
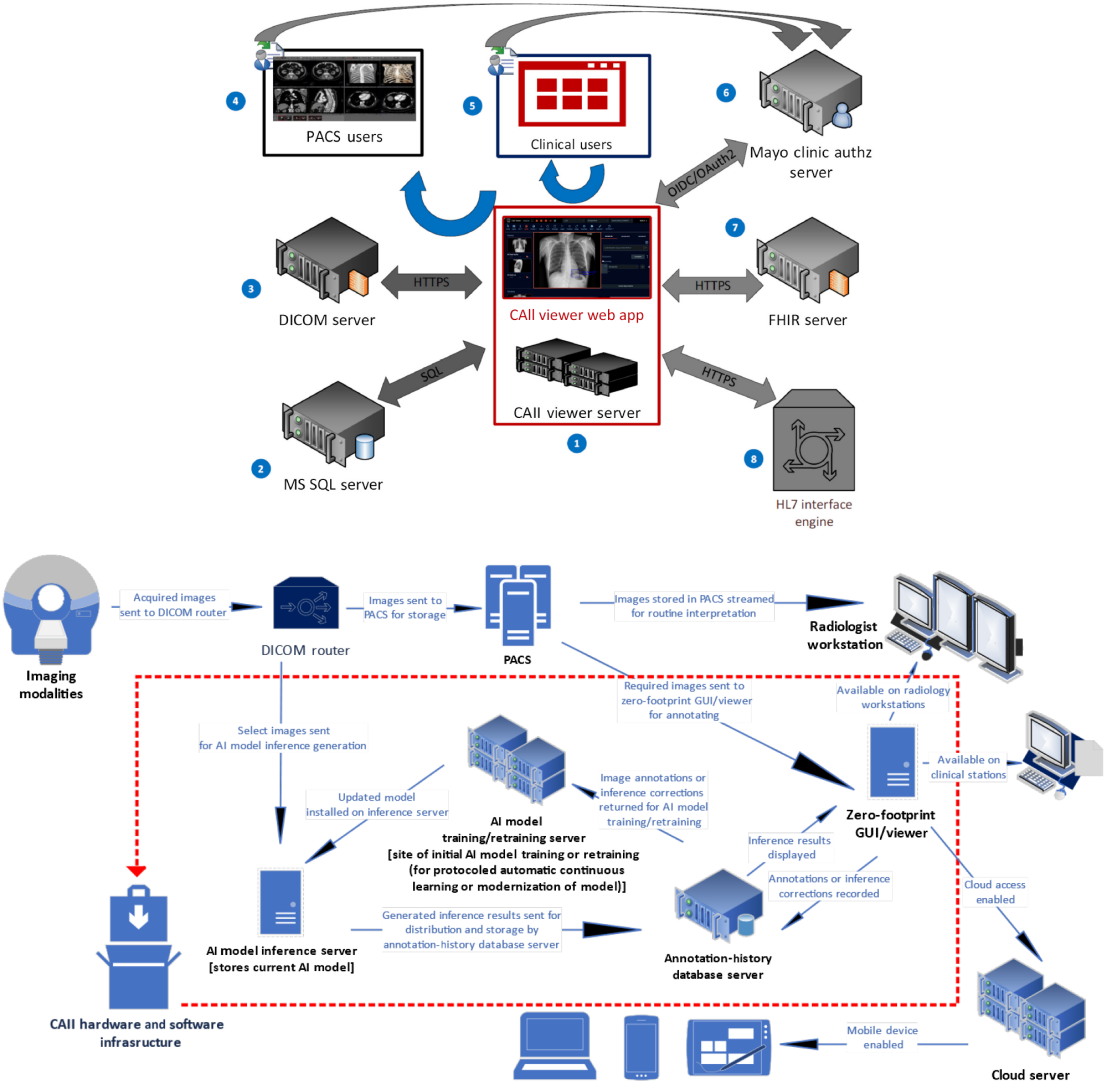
Development of viewer for model inference-result display and adjudication. As shown above, the developed ZF GUI/viewer: (1) Has basic functionalities under two main categories [(a) Server-level connectivity; (b) web-based user interface]; (2) Stores information about all image annotations, AI-model inferences, and user responses in an SQL server backend database; (3) Caches all images to be displayed to users in an Orthanc-based DICOM server; (4) Can be invoked from the PACS viewer via URLs passing image-specific parameters (e.g., accession numbers); (5) Can be summoned by clinical users via EMR systems by medical record numbers or accession numbers; (6) Facilitates user interactions, including single-sign-on logins enabled by authorization servers; (7) Supports FHIR interconnectivity (e.g., for placing order messages invoking model inference-result display on specific images); (8) Can absorb traditional HL7 order messages. As represented below, the ZF GUI/viewer is designed to be potentially integrated into the clinical PACS-support infrastructure.

Our deployment simulation initially relied on the previously described GUI^36^^,^^47^ for model inference-result display to the end-user [Table 3]. A zero-footprint (ZF) viewing platform (aka “ZF GUI/viewer”) has since been designed to support all phases of imaging-AI model development and evolution in a user-interactive fashion (Appendix B with Fig. 5).^43^^,^^66^^,^^67^

##### Applying data standards, supporting interoperability, and enhancing user experience

Whether or not inference results from an AI model provide helpful insights, it is crucial that end-users prospectively adjudicate (i.e., accept, modify, or reject) the results on a case-by-case basis to reinforce the essential adaptation of the AI model to changing real-world conditions.^40^^,^^41^^,^^44^^,^^45^^,^^67^^,^^68^ To that end, DICOM-structured reports (DICOM-SR) were recruited in the ZF GUI/viewer primarily for assigning spatial coordinates and simple shapes linked to coded text labels, all highly applicable to this work (Table 3; Appendix C).^42^^,^^43^^,^^69^^–^^71^

In addition, based on the practical experience of the ground-truth expert with the operations of the ZF GUI/viewer to date, noncontributing inference-display redundancy and complexity due to multiple overlapping identically labeled GBBs were reduced (Appendix C).

##### Preparation for Ongoing Adaptation of LLIED-Detection and Identification AI Model

A note about the basic pre-deployment trialing in the methodology trial population was that three new LLIED types (all LLRs) (Table 1 and Fig. 2) (not previously represented in the AI model development population and, consequently, not signified by classes in original LLIED model) were discovered. This unanticipated early prereal-world challenge created immediate demands to avoid the associated “concept drift”^45^^,^^46^ and to facilitate model adaptation^65^^–^^68^ prior to a true real-world deployment (Table 3). Our methodologic response was as follows.

With the need to supplement the number of cases of the three new LLIED types, as well as any of the original nine LLIED types, which were initially sparsely represented in the AI model development population, sequential patients with LLIED-demonstrating frontal CXRs accrued after the methodology trial population (i.e., July 2021 to February 2022) were inspected for additional examples.

These needed additional LLIED cases were annotated, as previously described. Their annotations were added, along with the corresponding LLIED annotations from the methodology trial population, to the already annotated CXR data from the AI model development population. As a result, a new and larger “AI model update population” was created to strengthen repeat training, validation, and testing of a new 12-class “updated LLIED model.” To that end, the same two-tier methodology was employed.^36^

#### 2.2.3 Performance evaluations of updated LLIED model for LLIED recognition

Performance results from the updated LLIED model were analyzed (Table 3), as previously described, including the following.

##### Cross-validation assessment

To assess durability of the updated LLIED model, a fivefold cross-validation^63^ was again executed on tier 1 for LLIED detection in the AI model update population (Table 3). However, as with the original LLIED model, lack of benefit from ROI pooling and significant data imbalance persisted, with some no-longer-implanted legacy LLIED types still represented by very small patient subsets (Fig. 4). Thus, meaningful cross-validation assessment of tier 2 accuracy in the identification of LLIED MRI-safety level and specific type could not be adequately evaluated.

##### Safety-level and specific-type identification accuracies during updated model testing

The specific identities, photographic appearances, CXR delineations, and MRI-safety levels of the three new classes, along with the original nine classes, of LLIEDs were tabulated (Table 1 and Fig. 2). The identification accuracy both per MRI-safety level (i.e., unsafe, stringently conditional, assessment-requiring conditional, or simple conditional) and per specific LLIED type, was re-evaluated in the AI model update population (Table 3). Again, the clinical MRI-safety implications of any model-related nondetections or misidentifications of LLIEDs were reviewed.

##### Limited-deployment simulated real-world trialing

To ensure maintenance of basic functionality of the updated two-tier cascading model with tier-1 LLIED detection and tier-2 LLIED identification, a limited-deployment (utilizing the ZF GUI/viewer functioning in our test clinical environment parallel with our routine workflow) allowed additional simulated real-world trialing (Table 3).

To mirror a real-world trialing of the updated LLIED model more closely, a subsequent “updated methodology trial population” representing a recent sequential series (spanning: February 2022 to June 2022) of 100 new LLIED-demonstrating frontal CXRs (not represented in prior described study populations) was analyzed, regardless of the specific LLIED type represented or the image quality demonstrated. In each case, simultaneously with the routine clinical CXR interpretation by the ground-truth expert, the frontal CXR suggesting the presence of an LLIED was processed prospectively within the parallel ZF GUI/viewer test environment using the 12-class updated LLIED model; the inference results were immediately expert-adjudicated for the presence/location (versus absence), as well as the type, of LLIED inferred. Concurrently interpreted clinical cases in which an LLIED was excluded (with or without inference-result adjudication against false-positive GBBs) were not included in the “updated methodology trial population.”

The compiled adjudication results were used to assess LLIED-identification accuracy by the 12-class updated LLIED model in the updated methodology trial population prior to true real-world deployment of our updated model and infrastructure architecture.

### 2.3 General Support of Methods

#### 2.3.1 AI technical infrastructure

All AI-model computations utilized several secure on-site graphics processing unit (GPU)-dependent systems. For training, validation, and testing of our AI models, an eight-GPU system [DGX A100 from Nvidia (Santa Clara, California)] was employed.

#### 2.3.2 Statistical analysis

As part of the standard analysis of testing results related to general LLIED detection in tier 1, precision–recall curves were plotted to reflect the basic comparison between the AI model output and ground-truth expert determinations.^36^^,^^72^ Tier-2 assessment of the discrimination performance of the multiclass AI model for LLIED-type identification used the area under the receiver operating characteristic curve (AUC ROC) methodology.^36^^,^^73^

## 3 Results

### 3.1 Performance Evaluations of Original LLIED Model for LLIED Recognition

#### 3.1.1 Cross-validation assessment

As previously reported (without disclosure of LLIED identities),^36^ tier 1 of the original LLIED model achieved the required 100% LLIED-detection sensitivity during testing.

**Fig. 6:**
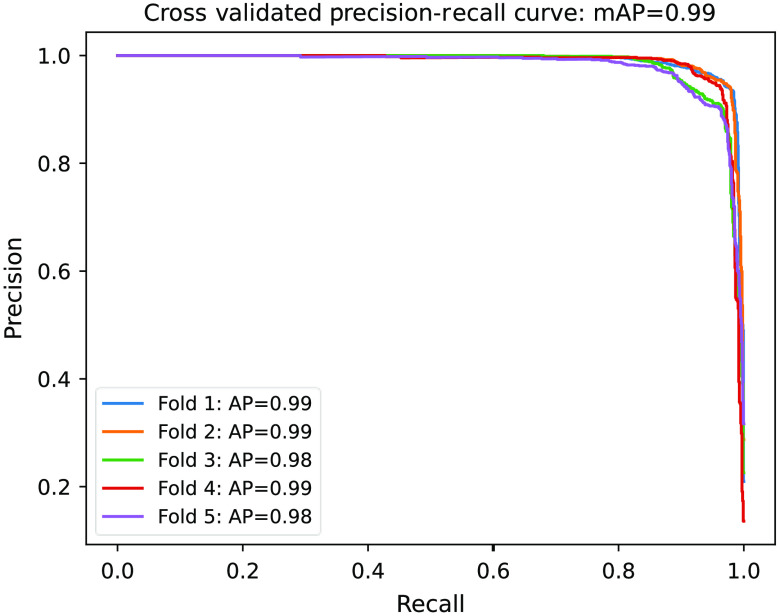
Precision–recall curves for two-class detection (tier 1) by original LLIED model.

In this work, during fivefold cross-validation, the mean average precision (mAP) was found to be 0.99 (Fig. 6), indicating the durability of the original LLIED model for LLIED detection and localization.

However, as previously mentioned, meaningful tier 2 cross-validation assessment of identification accuracies was precluded.

#### 3.1.2 Safety-level and specific-type identification accuracies during model testing

Also as previously described,^36^ tier 2 of the original LLIED model reached high generic performance levels for LLIED classification. Of those classified as LLIED types, the identification assignments were overall correct at 98.9% during model testing in the AI model development population.^36^

In this work, AUCs (rounded to nearest 1/100th) for identification of MRI-safety level category (i.e., unsafe, stringently conditional, assessment-requiring conditional, or simple conditional) consistently matched or exceeded 0.98, accompanied by high sensitivities (≥99%) and specificities (≥90%) (Table 4).

**Table 4:** Original LLIED model for safety-level identification—model testing.

Ground truth		Prediction							
ROI labels	# ROIs	Simple conditional (All LLRs)	Assessment- requiring Conditional (Both LLPs)	Stringently conditional (CardioMEMS HF)	Unsafe (BRAVO RC)	nonLLIED	SENSITIVITY	SPECIFICITY	AUC
**Simple conditional** (All LLRs)	484	480	3	1			0.99	0.98	0.98
**Assessment-requiring conditional** (Both LLPs)	334	1	329	4			0.99	0.93	1.00
**Stringently conditional** (CardioMEMS HF)	33			33			1.00	0.90	1.00
**Unsafe** (BRAVO RC)	27				27		1.00	1.00	1.00
nonLLIED	4481	106	370	522	21	3462	0.77	1.00	—

Identification accuracies for the original nine specific LLIED types were also high with AUC 1.00 for eight types and 0.92 for one LLR type (Table 8).

#### 3.1.3 Predeployent trialing

**Fig. 7:**
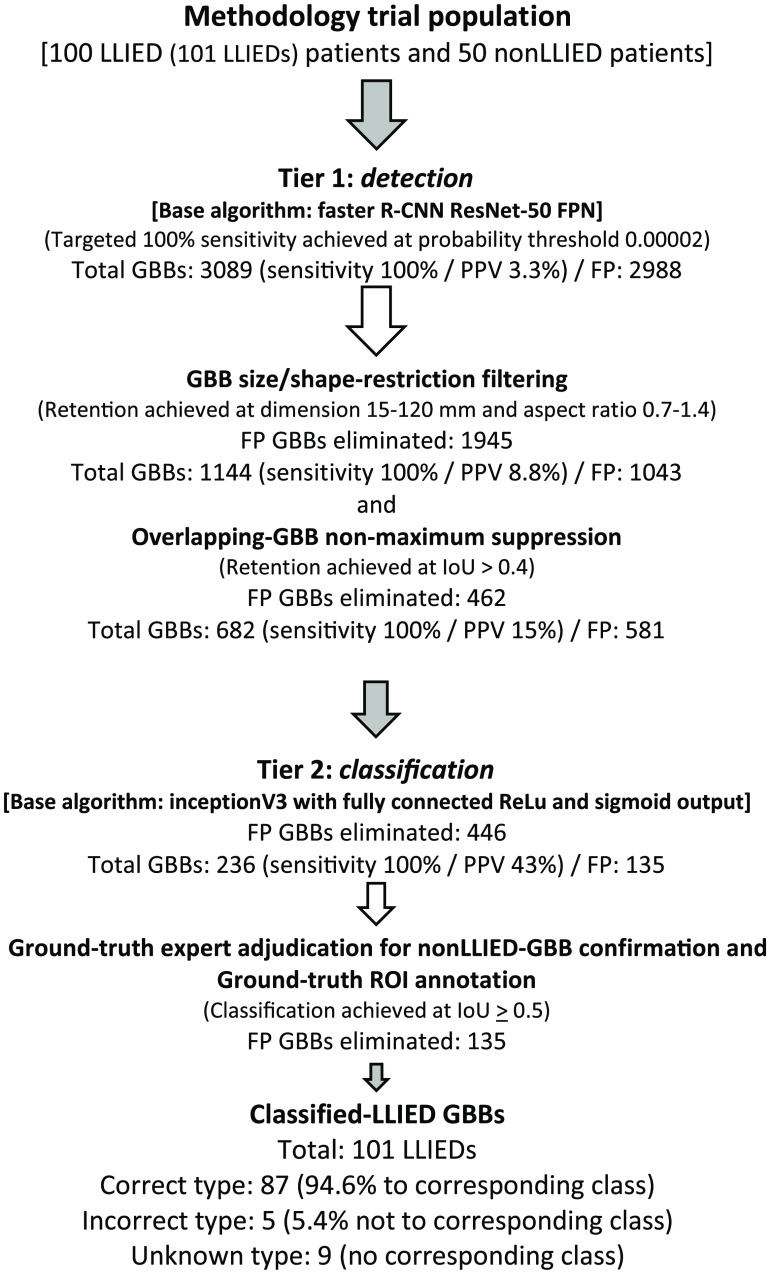
Basic pre-deployment trialing in methodology trial population. (FP, False positive; GBB, generated bounding box; IoU, intersection-over-union; PPV, positive predictive value).

Based on postinference ground-truth judgments, the results of the imitated basic real-world trialing experience in the 150 unannotated frontal CXRs from the methodology trial population were strong. They demonstrated the following: (1) maintained detection sensitivity of 100% at the temporary cost of increased GBBs (total 682) from tier-1 processing, with most FP GBBs immediately eliminated transparently by tier-2 processing (i.e., 446 of 682 GBBs excluded) and then the remaining via ground-truth adjudication of inference results (i.e., 135 displayed FP GBBs disqualified by end-user); (2) Ongoing high specific-type identification accuracy at 94.6% (87 of 92 LLIEDs) if preestablished corresponding classes were present at the time of tier-2 processing of the original LLIED model (Fig. 7).

Of the 101 LLIEDs represented in the methodology trial population, most with corresponding classes within the original LLIED model were correctly identified per safety-level category, with a high overall accuracy of 98% (99 of 100, with sensitivities, ≥95% and specificities ≥90%) (Table 5). Specific LLIED types were also identified with high accuracy (Table 9).

**Table 5:** Original LLIED model for safety-level identification—basic trialing.

Ground truth		Prediction						
ROI labels	# ROIs	Simple conditional (All LLRs)	Assessment- requiring conditional (Both LLPs)	Stringently conditional (CardioMEMS HF)	Unsafe (BRAVO RC)	nonLLIED	SENSITIVITY	SPECIFICITY
**Simple conditional** (All LLRs)	65	64		1			0.98	0.99
**Assessment-requiring conditional** (both LLPs)	22	1	21				0.95	0.91
**Stringently conditional** (CardioMEMS HF)	5			5			1.00	0.90
**Unsafe** (BRAVO RC)	9				9		1.00	1.00
nonLLIED	581	8	58	68	1	446	0.77	1.00

**Fig. 8:**
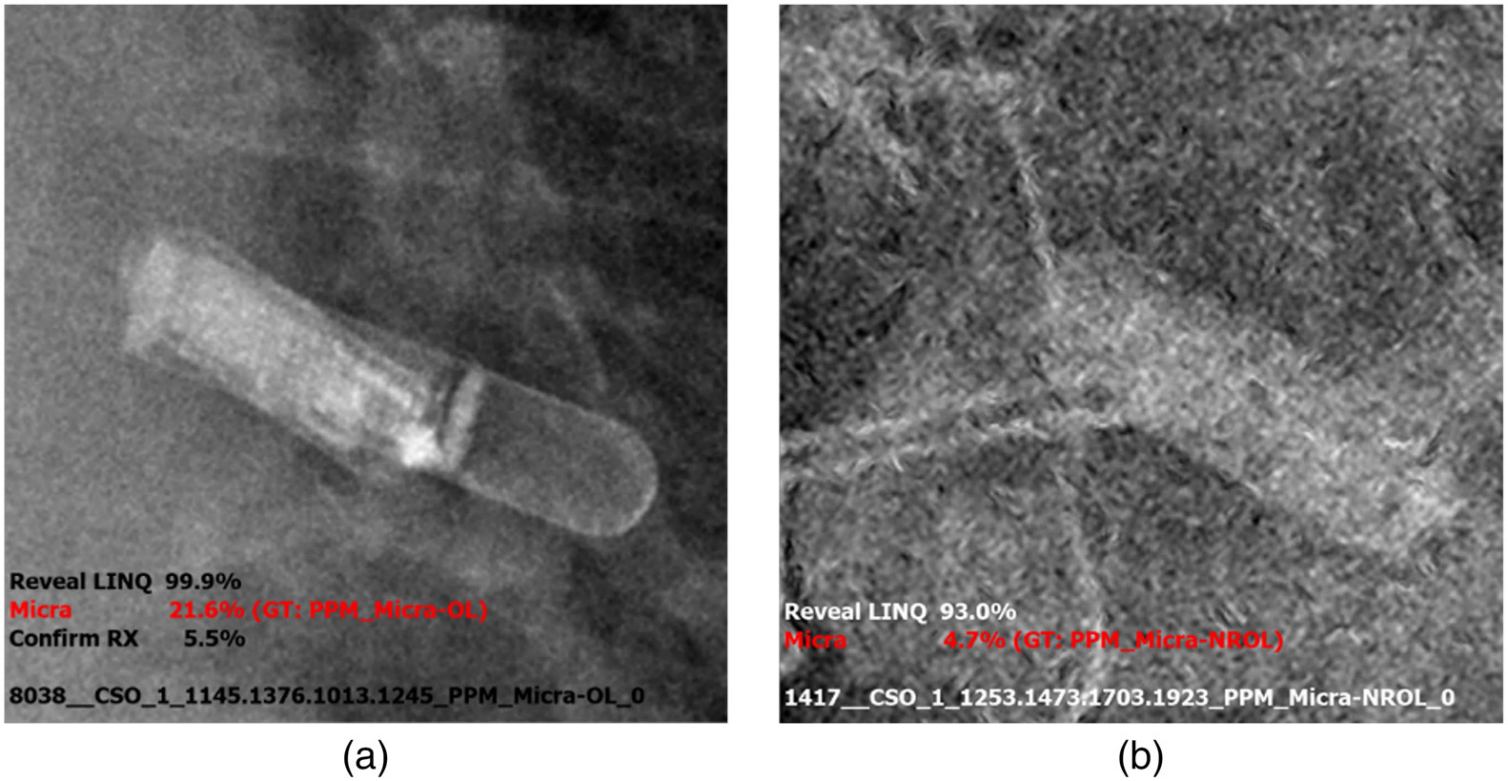
Misidentified LLPs by the original LLIED model due to suboptimal image quality. (a) During model testing (Table 8), an LLP misidentification was attributable to OL image quality with extraordinary superimposition of an LLR (i.e., Reveal LINQ™ identified with 99.9% probability) on the LLP (i.e., Micra™ identified next with 21.6% probability). (b) During basic trialing (Table 9), a misidentified LLP (i.e., Micra™ correctly identified with 4.7% probability, after a reveal LINQ™ identified with 99.9% probability) was attributable to NR/OL image quality related to poor general conspicuity and detail clarity, as well as to superimposed sternal wires.

**Fig. 9:**
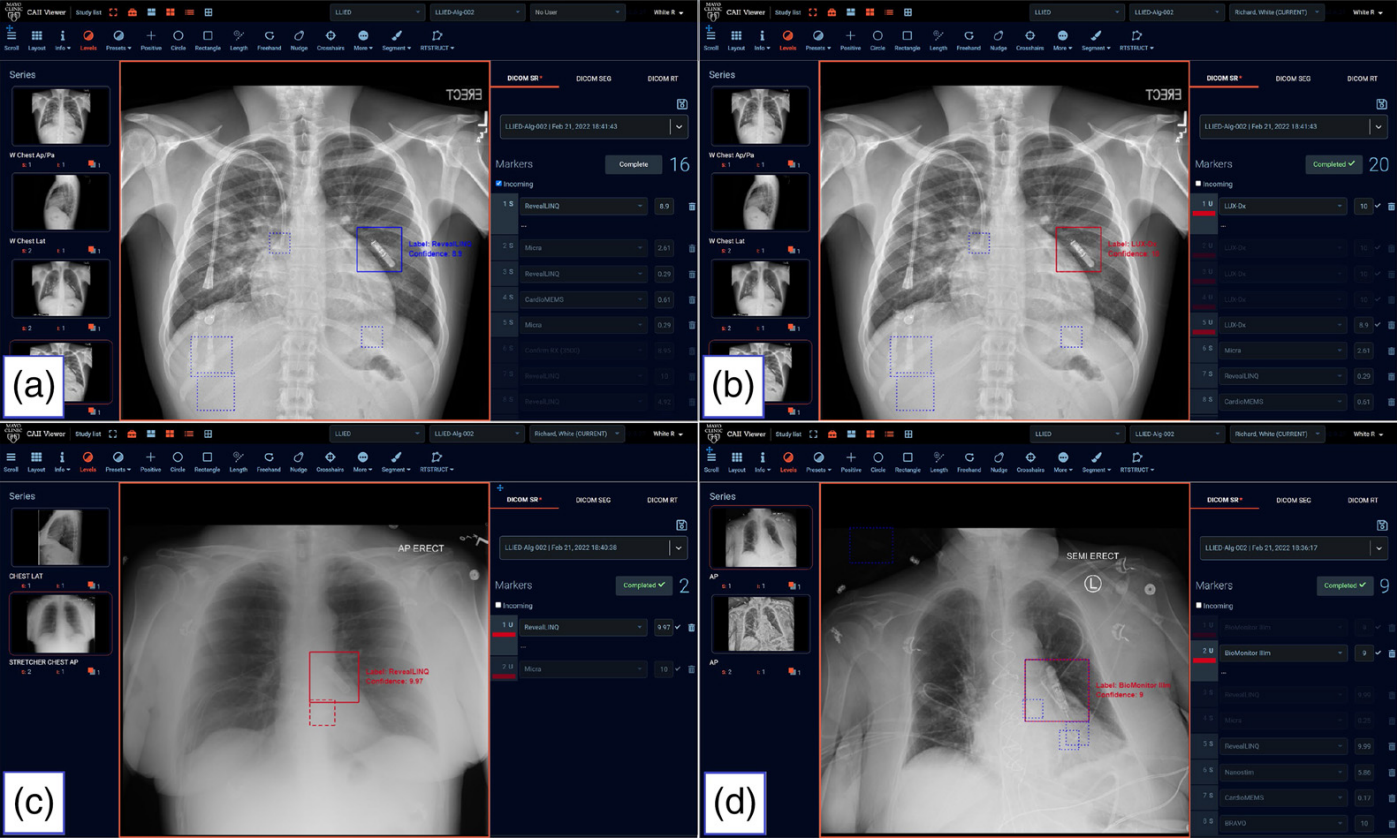
ZF GUI/viewer demonstrating inference results (location and probabilities) for end-user adjudication on three new LLIED types in methodology trial population. Previously, unclassified LLR types (i.e., A and B = LUX-Dx™; C = LINQ™ II; D = BioMonitor III) were properly detected as simple conditional LLRs by the original LLIED model, although misidentified as the most common LLR (i.e., Reveal LINQ™) of equal MRI safety (i.e., simple conditional). A previously classified assessment-requiring conditional LLP (i.e., C = Micra™) was both correctly detected and identified at a high probability level, with an appropriate inference GBB label automatically assigned. The user-friendly inference-adjudication capabilities of the ZF GUI/viewer allowed manual label reassignment of inference GBB labels from a drop-down list (e.g., Reveal LINQ™ in A relabeled as LUX-Dx™ in B), or confirmation of correct automatic assignment (e.g., Micra™ in C). In addition, an “other” option for labeling of false-negative or unanticipated future LLIEDs by the end-user is also included. All end-user adjudications of inference results are recorded in the ZF GUI/viewer backend database in support of model continuous learning and modernization.

Due to the 100% detection sensitivity achieved by tier 1 of the original LLIED model, no LLIEDs went undetected in the just-described experiences related to either the AI model development population or methodology trial population. However, of the cases misidentified when there were corresponding classes (10/878 = 1.1% of LLIED-related ROIs in AI model development population and 5/101 = 5.0% of LLIED-demonstrating frontal CXRs), the majority [11 of 15 = 73%, representing 8/10 cases and 3/5 cases, respectively (Tables 8 and 9)] could be attributed to suboptimal image-quality grades (cumulatively five NR and OL, four NR, and two OL) (Fig. 8). However, in the methodology trial population, the overall majority of misidentified cases (9 of 14 cases) were ascribed to prior absence of corresponding classes in the original LLIED model for the three new LLR types; this necessitated adjudication correction of the inference results by the ground-truth expert (Fig. 9) for future model modernization including the development of the needed new classes.

### 3.2 Performance Evaluations of Updated LLIED Model for LLIED Recognition

The AI model update population included new 351 frontal CXR examinations from 219 patients not previously included in the original smaller AI model development population to support the training, validation, and testing of the 12-class updated LLIED model. The previously reported technologic methodology for model development was re-used.^36^

#### 3.2.1 Cross-validation assessment

As with the original LLIED model, tier 1 of the updated LLIED model achieved 100% LLIED detection sensitivity during testing. During fivefold cross-validation, the mAP of the updated LLIED model was again 0.99, indicating its detection durability. However, as in the case of the original LLIED model, due to significant data imbalance (with some no-longer-implanted legacy LLIED types or new LLIED types still represented by very small patient subsets) meaningful cross-validation assessment of identification accuracies (safety-level or specific-type) could not be adequately evaluated.

#### 3.2.2 Safety-level and specific-type identification accuracies during updated model testing

Like with the nine-class original LLIED model in the AI model development population, tier-1 LLIED-detection of 100% was followed by high classification performance for LLIED identification by the 12-class updated LLIED model in the AI model update population, with the identification assignments overall correct at 99.5% during model testing.

AUCs for the identification of the category of MRI-safety level (i.e., unsafe, stringently conditional, assessment-requiring conditional, or simple conditional) consistently matched or exceeded 0.99, accompanied by high sensitivities (≥99%) and specificities (≥90%) [Table 6].

**Table 6:** Updated LLIED model for safety-level identification—model testing.

Ground truth		Prediction							
ROI labels	# ROIs	Simple conditional (All LLRs)	Assessment- requiring conditional (Both LLPs)	Stringently conditional (CardioMEMS HF)	Unsafe (BRAVO RC)	nonLLIED	SENSITIVITY	SPECIFICITY	AUC
**Simple conditional** (All LLRs)	552	549	2	1			0.99	0.95	0.99
**Assessment-requiring conditional** (both LLPs)	340		340				1.00	0.91	1.00
**Stringently conditional** (CardioMEMS HF)	35			35			1.00	0.90	1.00
**Unsafe** (BRAVO RC)	29				29		1.00	1.00	1.00
nonLLIED	5335	307	528	389	38	4073	0.76	1.00	—

For the identification of the original 9, plus three new, specific LLIED-types, AUCs were 1.00 for nine types, and 0.92 to 0.99 for three LLR types (Table 10).

Of the five misidentified LLR cases, the updated LLIED model displayed on the ZF GUI/viewer the correct label assignment as the second, third, and fourth most likely in 2, 2, and 1 case(s), respectively. Suboptimal image quality was applied to two (both NR) of the five misidentified cases.

#### 3.2.3 Limited-deployment simulated real-world trialing of updated LLIED model

The initial use of the ZF GUI/viewer in our near-real world clinical test environment, with its DICOM-SR output for this project, supports immediate model inference-result presentation (including 0% to 100.0% probability display) simultaneously with the CXR examination posting on the clinical PACS worklist. The previously described purposeful display-limitation of stacked overlapping and identically labeled inference-GBBs to the one GBB with the highest probability level on a case-by-case basis enhanced end-user experience by eliminating an extra 1 to 17 noncontributing overlapping identically labeled GBBs in 63 of the 100 cases. The result was a remarkably simpler inference-result adjudication process without loss of model performance.

**Fig. 10:**
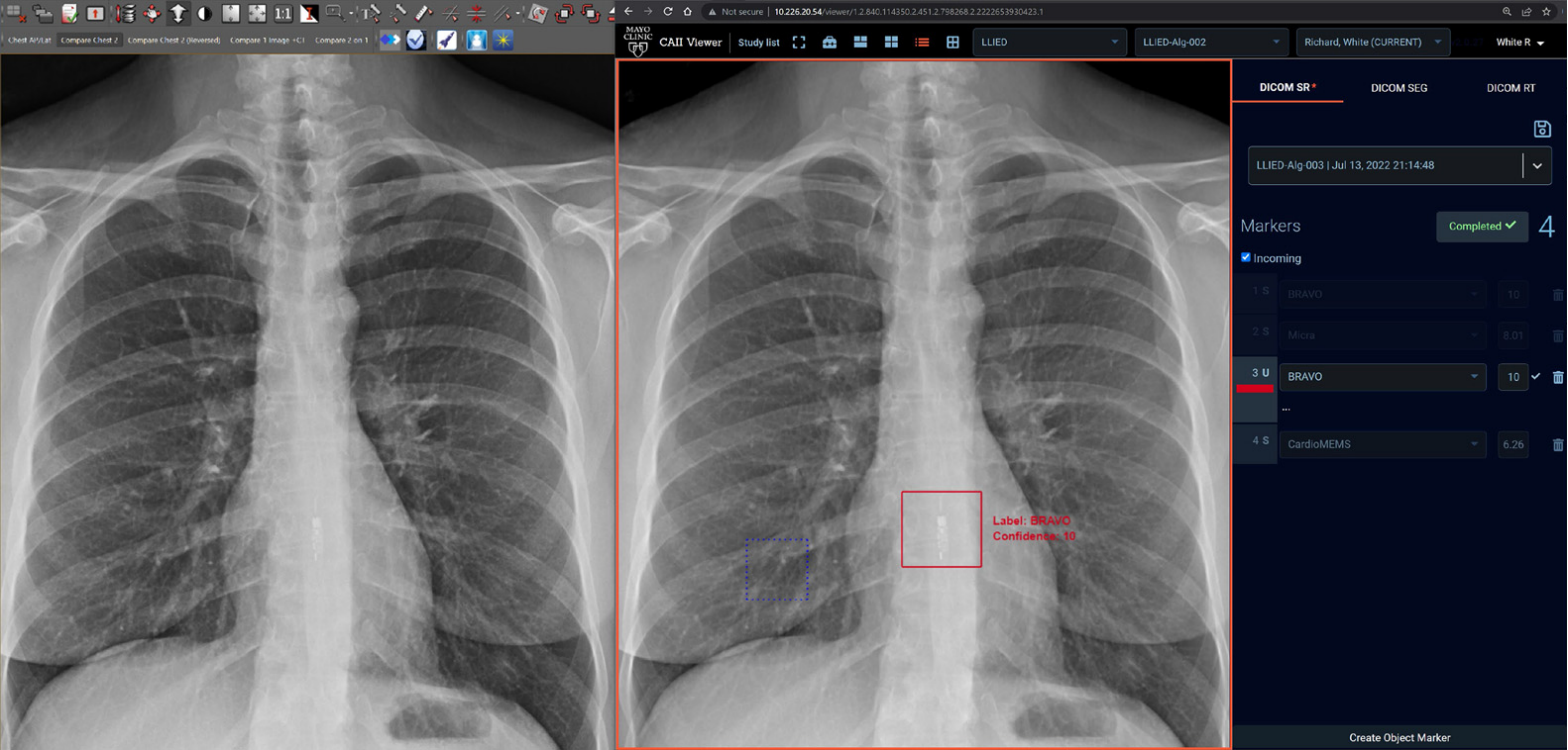
Simultaneous adjacent clinical PACS and ZF GUI/viewer displays. In the limited-deployment test environment, the standard clinical PACS display (a) is simultaneously accompanied by an adjacent display of inference results by the ZF GUI/viewer (b) on the same monitor used by radiologist for routine clinical image interpretations, although not yet integrated. On this frontal CXR image, the 12-class updated LLIED model has correctly detected/localized and identified (with GBB) the MRI-unsafe ERC (Bravo™ Reflux Capsule), with immediate single-click positive adjudication (Red boundaries applied with acceptance) versus passive rejection of any false-positive GBBs (maintained blue boundaries).

When combined, these capabilities facilitated user-friendly adjudication of inference results (by conventional clicking) within seconds, including (1) Acceptance of a result correctly identifying an LLIED; (2) Correction (relabeling) of a misidentified LLIED result; (3) Result rejection by simple passive disregarding of a false-positive nonLLIED GBB (Fig. 10).

The results of the prospective application of the 12-class updated LLIED model within the parallel ZF GUI/viewer test environment in the 100-case updated methodology trial population were also strong. Following tier-1 100% detection of all 101 LLIEDs (two LLIEDs in one case), strong tier-2 overall accuracy of 97% (98 of 101; sensitivities 75% to 98% and specificities 92% to 93%) for the identification of safety-level category was achieved; stringently conditional and unsafe categories were not represented in this experience (Table 7).

**Table 7:** Updated LLIED model for safety-level identification—limited deployment.

Ground truth		Prediction						
ROI labels	# ROIs	Simple conditional (All LLRs)	Assessment- requiring Conditional (Both LLPs)	Stringently conditional (CardioMEMS HF)	Unsafe (BRAVO RC)	nonLLIED	SENSITIVITY	SPECIFICITY
**Simple conditional** (All LLRs)	97	95	2				0.98	0.93
**Assessment-requiring conditional** (both LLPs)	4	1	3				0.75	0.92
**Stringently conditional** (CardioMEMS HF)							—	—
**Unsafe** (BRAVO RC)							—	—
nonLLIED	592	41	52	70	3	426	0.72	1.00

Specific LLIED types were also identified with a strong overall accuracy of 95% (96 of 101 LLIEDs, including all but 1 of the 14 examples of the three newly classified LLR types) (Table 11).

Of the five misidentified LLIED cases, a valid GBB displaying the correct LLIED-type label was shown by the ZF GUI/viewer as being the second most likely in two cases and third most likely in three cases. Suboptimal image quality was noted in three (two OL and one NR) of the five misidentified cases.

## 4 Discussion

The promise of AI to improve patient safety is well recognized.^74^ This study focused on gaining insights into the performance readiness, including potential safety implications, as well as essential infrastructural and operational requirements, of an AI model prior to its deployment for real-world application. Promoting improved pre-MRI safety screening,^4^^–^^14^^,^^17^^,^^18^ our LLIED methodology^36^ had been developed to potentially assist radiologists in reviewing digital frontal CXRs for the detection/localization and identification of a range of commonly inserted LLIEDs; they vary in (1) MRI-related safety; (2) Associated interservice support needs (i.e., cardiology peri-MRI evaluations of an assessment-requiring conditional LLP); (3) Related requirements for MRI scanning modifications (e.g., more basic scanning with a stringently conditional PAPM).

### 4.1 AI Model Performance Optimizations and Clinical Implications

#### 4.1.1 Uniqueness of LLIED use-case and developed AI model

The practical clinical use-case^65^^,^^66^ inspiring our initial development^36^ is distinctively different from the most closely corresponding pursuits,^30^^–^^35^ due to its focus on the continuously evolving array of modern much-smaller LLIEDs being inserted into the chest with greater frequency. To our knowledge, this is the first reported achievement of AI-based radiographic detection and identification (important to FDA recalls, such as the Nanostim LLP for dysfunction, as well as to MRI safety) directed at LLIEDs, ranging from MRI-conditional to MRI-unsafe.

From the beginning, this work emphasized real-world conditions^36^^–^^38^^,^^40^^,^^41^^,^^43^^–^^46^ by (1) Utilization of large datasets representing multiple geographically dispersed sites for model development; (2) Representation of varying general radiographic technology producing digital CXRs over almost three decades; (3) Inclusion of all LLIED image qualities (e.g., NR, OL, and NR and OL, cumulatively representing 24% and 30% of AI model development population and methodology trial population, respectively); (4) Model retraining to account for previously unclassified LLIED types (i.e., creating a newer 12-class updated LLIED model to replace the original nine-class model); (5) Simulation of initial real-world trialing of both LLIED AI models on separate patient series (i.e., methodology trial population and updated methodology trial population).

#### 4.1.2 LLIED detection/localization and identification performance of the adapting AI model

We found both the 9-class and 12-class LLIED AI models to consistently achieve the premandated 100% detection/location sensitivity (in tier 1) in all described pre-deployment experiences; the durability of the two models was confirmed by fivefold cross-validations. In addition, both models consistently achieved high identification accuracies (in tier 2) for MRI-safety category and specific-type in all reported evaluations, including mimicked real-world trialing (i.e., 98% and 97% correct safety-level categorizations in the methodology trial population and the updated methodology trial population, respectively).

#### 4.1.3 Clinical implications of the adapting LLIED model

Due to the strength of tier-1 processing in our cascading AI methodology, no LLIEDs went undetected in any of the described experiences with either the original LLIED model or the updated LLIED model. Tier 2 related misidentifications were uncommon and most often attributable to suboptimal image quality.

When misidentifications were considered from an MRI-safety standpoint, it was noted that in our reported cumulative pre-deployment experience, there were no cases of tier-1 nondetection and/or tier-2 misidentification of either an MRI-stringently conditional PAPM (i.e., CardioMEMS™ HF) or an MRI-unsafe ERC (i.e., Bravo™ Reflux Capsule), even when an LLIED type was not previously classified. Thus, end-user adjudication of displayed inference results on these two more risky LLIED categories/types was consistently positive, thereby fully supporting higher levels of awareness of greater potential MRI risk in affected patients.

In the presence of corresponding classes for tier-2 processing, only 21 instances of MRI-conditional LLIED misidentification were found in the following decreasing order: (1) Simple Conditional LLR misidentified as another LLR (eight instances); (2) Simple Conditional LLR over-identified as an assessment-requiring conditional LLP (i.e., Micra™) (seven instances); (3) Simple conditional LLR over-identified as a stringently conditional PAPM (i.e., CardioMEMS™ HF) (three instances); (4) Assessment-requiring conditional LLP (i.e., Micra™) under-identified preadjudication as a simple conditional LLR (three instances). Respectively, the related potential clinical safety and operational implications included (1) No negative impact; (2) Premature operational considerations (e.g., unnecessary engagement of cardiology for peri-MRI assessments); (3) Premature safety considerations (e.g., plans to over-emphasize more basic forms of scanning); (4) Initial underestimation of needed coordination of operational support (e.g., failure to engage cardiology for needed peri-MRI assessments). However, it is important to realize that, as a decision-support assistant, the inference results generated by our LLIED methodology (with 100% LLIED detection/localization) are displayed directly to the radiologist for their adjudication before clinical use. Therefore, such inconsistencies are likely temporary and become corrected during the regular workflow, which is designed to actively involve the radiologist (rather than to function autonomously) and, hopefully in the future, is enhanced through integration with the EMR.

### 4.2 Essential Architectural, Workflow, and User-Experience Preparations and/or Enhancements

Repetitive updating of an already mature and deployed AI model used in healthcare settings has become a major focus of the AI community.^44^ This goal is highly dependent upon real-world experiences with the clinical application of AI models,^37^^,^^38^^,^^46^ requiring periodic model retraining to account for insights from end-user adjudications of model inference results.^40^^,^^43^ The needed feedback to the models facilitates “concept drift” avoidance^45^^,^^46^ and ongoing adaptation,^65^^–^^68^ hopefully resulting in more robust and improved future performance.^44^^,^^67^

The importance of such continuous learning was reinforced in our pre-deployment work by the fact that LLIED misidentifications were most often related to suboptimal image quality, followed by the appearance of new and previously unclassified types. Nevertheless, in our proposed clinical implementation, relying on the user-friendly ZF GUI/viewer created in response, all LLIED cases would be detected by our two-tier cascading AI model (delineated by a GBB) regardless of image quality, thereby already assisting the radiologist in LLIED recognition prior to adjudication of the displayed identification labels with simple click-based responses for model retraining. If a new LLIED type is recognized during routine clinical work, an ROI with a label (generic or specific) can be easily applied by the radiologist for model updating.

Less often acknowledged than continuous learning, but just as pertinent, is the need to keep an AI model modernized,^45^^,^^46^ thereby making it more resistant to “catastrophic forgetting.”^45^^,^^75^ This work revealed the urgency to accommodate the unexpected early appearance of three new LLIED types, necessitating pre-deployment retraining of the original nine-class AI model without loss of original classification capabilities, thereby creating a fully functional 12-class AI model more ready for deployment.

Our goal is to operationalize the aforementioned continuous-learning and modernization processes, when needed (e.g., per number of user experiences, deployment time, added new devices, or CXR data sources), relying on the backend database capabilities currently supporting the ZF GUI/viewer with essential real-time monitoring and recording of all interactions with the system.^41^^,^^44^^,^^76^^,^^77^ To our knowledge, beyond conceptual descriptions,^41^^,^^45^^,^^46^^,^^68^^,^^75^ there have been no other academic or commercial reports of standard processes designed to support such combined continuous learning/modernizing of imaging-AI models.

### 4.3 Limitations

We recognize the following limitations of our study.

First, the current need to execute our cascading models at a very low probability threshold to prevent LLIED-detection failure creates additional GBBs, resulting in (1) additional FP GBBs per correctly detected implant (i.e., TP result) in an LLIED case; (2) FP GBBs suggesting the presence of LLIEDs in a nonLLIED case.^36^ Future considerations for dealing with this limitation include (1) Adjustment of model parameters based on overall case-by-case CXR image quality;^36^ (2) Application of a single-tier faster R-CNN for blending detection and identification.^78^^,^^79^

Second, while this work represents the experience of a single-institution with inherent population bias (although LLIED designs are fixed according to FDA regulations), the input data represented many sites (∼75) distributed nationwide, which contributed many years-worth (over 30) of digital CXR data.^36^ Nevertheless, with our populations representing inflated LLIED prevalence, our reported model performances were potentially positively impacted.^72^^,^^73^ We plan to rely on insights from postdeployment experience to guide future retraining needs.

### 4.4 Future Directions

The next phase of this work will focus on a true deployment of the described methodology, (including 12-class updated LLIED model, ZF GUI/viewer, and repetitive continuous learning/modernization-based model retraining) within an appropriate clinical setting (e.g., for prospective “real-world performance” monitoring and with a “predetermined change control plan”).^44^ Additional output considerations include (1) Engagement incentives to radiologists for adherence to adjudication activities (e.g., complimented by learning experiences worthy of CME crediting); (2) Full integration of the LLIED model and ZF GUI/viewer into standard RIS-PACS configurations; (3) Direct transmission of adjudicated LLIED results to designated fields in CXR reports or patient EMRs,^80^ thereby reducing the chances for recording errors.^15^

## 5 Conclusion

This work assessed a previously described imaging-AI model during a pre-deployment exercise, which provided the following important insights: (1) Robust 100% detection sensitivity for general LLIED presence/location by both the original nine-class model and a newer 12-class model is achieved during model testing and simulated real-world trialing; (2) High identification accuracies for LLIED safety-level and specific-type are concurrently achieved by the same models; (3) Both versions of the basic LLIED model consistently and correctly detect and identify stringently MRI-conditional and MRI-unsafe types of LLIEDs; (4) Continuous learning and/or updating of the basic LLIED model are essential processes that were both demonstrated due to the early appearance of LLIED types; (5) A user-friendly ZF GUI/viewer, created to meet anticipated inference-result display and adjudication needs, is vital to a successful imaging-AI model deployment and facilitation of radiologist engagement. Of course, the actual value of our methodology will need to be assessed during a true real-world deployment in an appropriate clinical setting.

## 6 Appendices

This section is intended for providing further information on our design strategies for clinical deployment as well providing further details on real-world algorithmic performance.

### 6.1 Appendix A: Essential Technical Developments Supporting Real-World Model Deployment and Adaptation

A component-based simulation of deployment of our methodology was considered consistent with several recent FDA-endorsed actions,^44^ including: (1) “predetermined change control plan” (e.g., algorithm change protocol for how a model will learn and change while remaining safe and effective); (2) “real-world performance” monitoring (e.g., seamless gathering and validation of relevant “real-world” parameters and ongoing collection of performance data). To these ends, such implementation must fully incorporate verified AI model output while presenting the inference results in a meaningful and highly user-friendly fashion (e.g., rapid return of results, uncomplicated display), thereby facilitating their utilization by the CXR-interpreting radiologist as deemed ethical, appropriate, and beneficial to patients.^40^^–^^44^^,^^65^^,^^66^

### 6.2 Appendix B: Selection/Development of Viewer for AI Model Inference-Result Display and Adjudication

Our custom-designed, flexible (on-prem or web-accessed) ZF GUI/viewer, potentially for future integration into the organization-wide clinical PACS-support infrastructure (Fig. 5),^43^ was created for a model inference-result display to the end-user, replacing our previously described GUI.^36^^,^^47^ The ZF GUI/viewer has been designed to support all phases of imaging-AI model development and evolution in a user-interactive fashion, including the following: (1) Basic image display; (2) Image annotation for input into model development; (3) Presentation of geographically coordinated model inference results in a conventional format (in <1  s); (4) Easy indication of ground-truth judgment and/or modification of inference results by the end-user for continuous feedback toward future model adaptation and hopefully improvement.^43^^,^^66^^,^^67^

### 6.3 Appendix C: Applying Data Standards, Supporting Interoperability, and Enhancing User Experience

It is crucial that end-users prospectively adjudicate (i.e., accept, modify, or reject) the inference results from an AI model on a case-by-case basis to reinforce the essential adaptation of the AI model to changing real-world conditions.^40^^,^^41^^,^^44^^,^^45^^,^^67^^,^^68^ This expectation dictated to us the need to utilize standards for variable data input and output to facilitate AI-model evolution. To that end, DICOM-structured reports (DICOM-SR) were recruited in the ZF GUI/viewer primarily for assigning spatial coordinates and simple shapes linked to coded text labels.^42^^,^^43^^,^^69^^–^^71^ DICOM-segmentation (DICOM-SEG) was also incorporated for future pursuits needing representation of more complex 3D shapes with the flexibility for manual editing during the adjudication process.^42^^,^^43^^,^^69^^–^^71^

Based on practical experience of the ground-truth expert with the operations of the ZF GUI/viewer, noncontributing inference-display redundancy and complexity (i.e., LLIED visualization hindered due to multiple overlapping identically labeled GBBs) was reduced via case-by-case limitation of the stacked inference-GBB display for each identified LLIED type to the one GBB with the highest probability level.

With incorporation of the aforementioned cumulative capabilities and user-experience enhancements (Appendices A and B), the ZF GUI/viewer is currently functioning in real-time with limited-deployment in parallel with the routine PACS-dependent workflow within a test clinical environment.^76^ This allows the radiologist, during routine clinical duties, to prospectively: (1) Apply any appropriate AI-model to CXRs; (2) Adjudicate returned inference results; (3) Identify needed modification of a model, the supporting architecture and/or workflow operations. The ZF GUI/viewer design incorporates DICOM-SR and DICOM-SEG formats to meet current and future needs for inference-result display and adjudication (e.g., relabeling and segmentation modification).^76^

**Table 8:** Original LLIED model for specific-type identification—model testing.

Ground truth		Prediction												
ROI labels	# ROIs	BioMon2-AF	Nanostim	Confirm Rx	Confirm	Reveal XT	CardioMEMS HF	BRAVO RC	Micra	Reveal LINQ	nonLLIED	SENSITIVITY	SPECIFICITY	AUC
**BioMonitor2-AF**	1	1										1.00	1.00	1.00
**Nanostim**	2		2									1.00	0.99	1.00
**Confirm Rx**	2			1						**1**		0.50	1.00	0.92
**Confirm**	5				5							1.00	1.00	1.00
**Reveal XT**	35					34			*1*			0.97	1.00	1.00
**CardioMEMS HF**	33						33					1.00	0.90	1.00
**BRAVO RC**	27							27				1.00	1.00	1.00
**Micra**	332						4		327	***1***		0.98	0.93	1.00
**Reveal LINQ**	441						*1*		*2*	438		0.99	0.98	1.00
nonLLIED	4481	0	32	1	1	1	522	21	338	103	3462	0.77	1.00	**—**

The confusion matrix from testing of the original LLIED model in the AI model development population indicates high accuracy for the identification of specific LLIED types, with AUC<1.00 in only one of the original nine LLIED types (i.e., confirm Rx LLR). Ten MRI-conditional LLIED cases, including five LLRs and five LLPs were misidentified. One simple conditional LLR was misidentified as another simple conditional LLR (bold). Four simple conditional LLR cases (italic) were over-identified, from an MRI-safety standpoint, as either assessment-requiring conditional LLPs (i.e., Micra™) in three cases or a stringently conditional PAPM (i.e., CardioMEMS™ HF) in one case. Similarly, four assessment-requiring conditional LLP cases (i.e., Micra™) (underline) were over-identified as stringently conditional PAPMs (i.e., CardioMEMS™ HF), and a fifth LLP case was under-identified preadjudication (bold-italic) as the most common simple conditional LLR, likely due suboptimal image quality [shown in Fig. 8(a)].

**Table 9:** Original LLIED model for specific-type identification—basic trialing.

Ground truth		Prediction											
ROI labels	# ROIs	BioMon2-AF	Nanostim	Confirm Rx	Confirm	Reveal XT	CardioMEMS HF	BRAVO RC	Micra	Reveal LINQ	nonLLIED	SENSITIVITY	SPECIFICITY
**BioMonitor2-AF**												—	—
**Nanostim**												—	—
**Confirm Rx**	7			5						**2**		0.71	1.00
**Confirm**	1					**1**						0.00	1.00
**Reveal XT**												—	—
**CardioMEMS HF**	5						5					1.00	0.90
**BRAVO RC**	9							9				1.00	1.00
**Micra**	22								21	***1***		0.95	0.91
**Reveal LINQ**	48						*1*			47		0.98	0.98
**New LLIED Types**	9									**9**		—	—
nonLLIED	581		2		1		68	1	56	7	446	0.77	1.00

The confusion matrix from the imitated basic real-world trialing of the original LLIED model in the methodology trial population indicates high accuracy for the identification of the original nine specific LLIED types. Twelve simple conditional LLR cases, including nine representing three new and previously unclassified LLIED types (“new LLIED types”), were misidentified as other simple conditional LLRs (bold). One simple conditional LLR was over-identified as a stringently conditional PAPM (i.e., CardioMEMS™ HF) (italic). One assessment-requiring conditional LLP (i.e., Micra™) was under-identified preadjudication (bold-italic) as the most common simple conditional LLR, likely due suboptimal image quality [shown in Fig. 8(b)].

**Table 10:** Updated LLIED model for specific-type identification—model testing.

Ground truth		Prediction															
ROI labels	# ROIs	BioMon2- AF	Nanostim	Confirm Rx	Confirm	Reveal XT	CardioMEMS HF	BRAVO RC	Micra	Reveal LINQ	LINQ II	LUX- Dx	Bioonitor III	nonLLIED	SENSITIVITY	SPECIFICITY	AUC
**BioMonitor2-AF**	2	1							*1*						0.50	1.00	0.92
**Nanostim**	2		2												1.00	1.00	1.00
**Confirm Rx**	3			2								**1**			0.67	1.00	0.97
**Confirm**	5				5										1.00	1.00	1.00
**Reveal XT**	35					34				**1**					0.97	0.99	1.00
**CardioMEMS HF**	35						35								1.00	0.94	1.00
**BRAVO RC**	29							29							1.00	0.99	1.00
**Micra**	338								338						1.00	0.91	1.00
**Reveal LINQ**	486								*1*	485					1.00	0.96	1.00
**LINQ II**	1										1				1.00	1.00	1.00
**LUX-Dx**	7						*1*					6			0.86	1.00	0.99
**BioMonitor III**	13												13		1.00	1.00	1.00
nonLLIED	5335	1	9		7	42	389	38	519	241	1	12	3	4073	0.76	1.00	**—**

The confusion matrix from testing of the updated LLIED model in the AI model update population indicates high accuracy for the identification of the 12 specific LLIED types, with AUCs <1.00 in only three (including two of the original nine) LLIED types. Five simple conditional LLR cases were misidentified, including two misidentified as another simple conditional LLR (bold). The other three misidentified simple conditional LLRs (italic) were over-identified, from an MRI-safety standpoint, as either an assessment-requiring conditional LLP (i.e., Micra™) in two cases or a stringently conditional PAPM (i.e., CardioMEMS™ HF) in one case.

**Table 11:** Updated LLIED model for specific-type identification—limited deployment.

Ground truth		Prediction														
ROI labels	# ROIs	BioMon2- AF	Nanostim	Confirm Rx	Confirm	Reveal XT	CardioMEMS HF	BRAVO RC	Micra	Reveal LINQ	LINQ II	LUX- Dx	Bioonitor III	nonLLIED	SENSITIVITY	SPECIFICITY
**BioMonitor2-AF**															—	—
**Nanostim**															—	—
**Confirm Rx**															—	—
**Confirm**															—	—
**Reveal XT**															—	—
**CardioMEMS HF**															—	—
**BRAVO RC**															—	—
**Micra**	4								3	***1***					0.75	0.92
**Reveal LINQ**	83								*2*	81					0.98	0.94
**LINQ II**	2			**1**						**1**					0.00	1.00
**LUX-Dx**	11											11			1.00	1.00
**BioMonitor III**	1												1		1.00	1.00
nonLLIED	592					2	70	3	52	35		2	2	426	0.72	1.00

The confusion matrix from the simulated real-world trialing of the Updated LLIED Model during limited-deployment in the updated methodology trial population indicates high accuracy for the identification of the 12 specific LLIED types. Two simple conditional LLR cases were misidentified as another simple conditional LLR (bold); two other LLR cases were over-identified, from an MRI-safety standpoint, as assessment-requiring conditional LLPs (i.e., Micra™) (italic). One assessment-requiring conditional LLP (i.e., Micra™) was under-identified preadjudication (bold-italic) as a simple conditional LLR.
